# Genomic profiling of Chinese patients with urothelial carcinoma

**DOI:** 10.1186/s12885-021-07829-1

**Published:** 2021-02-15

**Authors:** Bo Yang, Xiao Zhao, Chong Wan, Xin Ma, Shaoxi Niu, Aitao Guo, Jieli Wang, Jinliang Wang, Decong Sun, Shunchang Jiao

**Affiliations:** 1 Department of Oncology, Chinese PLA General Hospital, Fuxing Road 28, Beijing, China; 2 Lifehealthcare Clinical Laboratories, Hangzhou, China; 3 Department of Urology, Chinese PLA General Hospital, Beijing, China; 4 Department of Pathology, Chinese PLA General Hospital, Beijing, China

**Keywords:** Urothelial carcinoma, Genomic alterations, ctDNA

## Abstract

**Backgrounds:**

Urothelial carcinoma (UC) is the most common genitourinary malignancy in China. In this study, we surveyed the genomic features in Chinese UC patients and investigated the concordance of genetic alterations between circulating tumor DNA (ctDNA) in plasma and matched tumor tissue.

**Materials and methods:**

A total of 112 UC patients were enrolled, of which 31 were upper tract UC (UTUC) and 81 were UC of bladder (UCB). Genomic alterations in 92 selected genes were analyzed by targeted next-generation sequencing.

**Results:**

In the study cohort, 94.64, 86.61 and 62.50% of patients were identified as having valid somatic, oncogenic and actionable somatic alterations, respectively. The most frequently altered genes included *TP53*, *KMT2D*, *KDM6A*, *FAT4*, *FAT1*, *CREBBP* and *ARID1A*. The higher prevalence of *HRAS* (22.0% vs 3.7%) and *KMT2D* (59.26% vs 34.57%) was identified in UTUC than in UCB. Comparisons of somatic alterations of UCB and UTUC between the study cohort and western cohorts revealed significant differences in mutant prevalence. Notably, 28.57, 17.86 and 47.32% of the cases harbored alterations in FGFRs, ERBBs and DNA damage repair genes, respectively. Furthermore, 75% of the patients carried non-benign germline variants, but only two (1.79%) were pathogenic. The overall concordance for genomic alterations in ctDNA and matched tumor tissue was 42.97% (0–100%). Notably, 47.25% of alterations detected in ctDNA were not detected in the matched tissue, and 54.14% of which were oncogenic mutations.

**Conclusions:**

We found a unique genomic feature of Chinese UC patients. A reasonably good concordance of genomic features between ctDNA and tissue samples were identified.

**Supplementary Information:**

The online version contains supplementary material available at 10.1186/s12885-021-07829-1.

## Background

Urothelial carcinoma (UC) is the most common genitourinary malignancy in the world [1]. As reported by the Chinese national cancer center, the estimated number of newly-diagnosed and mortality of urothelial carcinoma cases in 2015 was 80,500 and 32,900, respectively [2]. Most non-muscle invasive bladder cancer patients have disease recurrence and progression to the invasive or metastatic disease within 5 years [3]. Treatments for locally advanced or metastatic disease have changed dramatically in the last 3 years, especially with the approval of the five immune checkpoint inhibitors (ICIs) and an FGFR inhibitor by the U.S. Food and Drug Administration (FDA).

Previous studies have comprehensively investigated the genomic features of urothelial carcinoma, but the samples were mainly from Caucasian patients [4]. To date, few studies had characterized the molecular features of Chinese UC patients. The genomic alterations in western and Chinese UC patients may be distinct, especially considering the the genetic backgrounds’ differences and the exposure to aristolochic acid in traditional Chinese herbal medicine [5]. Meanwhile, as most of the previous genomic research in UC was conducted on the tumor tissues, patients without sufficient tumor samples or those who were unable to undergo biopsy will miss the chance of target therapies. Recently, circulating tumor DNA (ctDNA) has been applied in cancer diagnosis, treatment selection and monitoring, especially for lung cancer. For localized bladder cancer, ctDNA in plasma and urine had been detected even at the early phase of disease, and correlated with disease recurrence or progression [6]. However, whether ctDNA in blood or urine would be a valid substitution for tissue testing in UC is still unknown.

In the present study, we analyzed the germline and somatic gene alterations in 112 Chinese UC patients. The basic genomic alteration profiles were described and compared with corresponding Western data. Moreover, we compared the concordance of genetic alterations between ctDNA in plasma and matched tumor tissue to evaluate the utility of liquid biopsy samples in UC patients.

## Methods

### Samples

This study’s protocol was approved by the ethics committee of the Chinese PLA General Hospital (S2019–302-01), and all enrolled patients have signed the informed consent. Formalin-fixed, paraffin-embedded (FFPE) tumor tissues and matched blood samples in EDTA tubes (for germline tests) from 112 diagnosed UC patients (Supplemental Table 1) were collected. All tumor FFPE sections were evaluated by pathologist to contain at least 20% tumor cells. Thirty-four patients who cannot provide sufficient or valid tumor tissue samples were collected plasma for ctDNA testing instead. Among patients who provided valid FFPE samples, 20 agreed to provide additional blood samples in Streck tubes to compare the genomic differences between Cell-free DNA (cfDNA) and matched tumor.

### DNA isolation

The FFPE tissues and peripheral blood mononuclear cells were collected to extract DNA using QIAamp DNA FFPE Tissue Kit and DNeasy Blood & Tissue Kit (Qiagen, Inc.), respectively. Cell-free DNA (cfDNA) was extracted from plasma using the QIAamp Circulating Nucleic Acid Kit (Qiagen, Inc.). The purified gDNA and cfDNA were quantified using the Qubit 3.0 Fluorometer (Life Technologies, Inc.) and Ste. pOnePlus System (Life Technologies, Inc.).

### Targeted next-generation sequencing

For the matched germline and tumor samples, 100 ng of DNA was sheared with a Covaris E210 system (Covaris, Inc.) to obtain an average of 200 bp fragments. Accel-NGS 2S DNA Library Kit (Swift Biosciences, Inc.) and xGen Lockdown Probes kit (IDT, Inc.) were used for NGS library preparation of the tumor gDNA matched germline gDNA. The custom xGen Lockdown probe was synthesized by IDT, Inc. to target the exons and selected intronic regions of 92 genes (Supplemental Table 2). The prepared library was quantified by using the Qubit 3.0 Fluorometer (Life Technologies, Inc.), and the quality and fragment size were measured by using Agilent 2100 Bioanalyzer (Agilent Technologies, Inc.). The libraries were loaded onto an Illumina Nextseq CN500 platform (Illumina Inc) for paired-end sequencing with a 150-bp read length. Mean coverage of 1260.5×, 3759.3× and 223.6 × were achieved for tumor gDNA, blood cfDNA and peripheral blood mononuclear cells gDNA, respectively.

### Data processing

Raw sequencing data were aligned to the reference human genome (UCSC hg19) by Burrows-Wheeler Aligner. After duplicate removal and local realignment, we applied Genome Analysis Toolkit (GATK) to identify single nucleotide variation (SNV), insertion and deletion (inDel). Next, after removing the germline alterations from matched blood samples, the somatic alterations were obtained. Variants were annotated using the ANNOVAR software tool. Copy number variations were analyzed using CNVkit. Genomic alterations data from The Cancer Genome Atlas database (TCGA) and Memorial-Sloan Kettering Cancer Center (MSKCC) database was downloaded from cBioPortal (http://www.cbioportal.org).

### Statistical analysis

Statistical analyses were performed using the Statistical Package for the Social Sciences (SPSS) statistical package and Graphpad (Prizm 8). Comparisons were conducted by utilizing the Fisher Exact test. Differences were considered significant if *p* < 0.05.

## Results

### Demographic and clinical data

The demographic and clinical data of patients enrolled in our cohort were summarized in Table 1. Of the enrolled patients, 72.32% had UC of bladder cancer (UCB, 81/112), and the rest were diagnosed as having upper tract UC (UTUC, 27.68%, 31/112), including carcinoma of the renal pelvis and ureteral. The median age at diagnosis was 67 (range 25–86). The sex ratio between males and females was 2.61:1, which was close to the sex ratio (2.97:1) reported by the Chinese national cancer center. The equal number of smokers and non-smokers were enrolled (56 versus 56).

**Table 1:** Demographic and clinical data of patients

Characteristics	*n*=112
Median age (range)	67 (25-86)
Sex	
Male	81
Female	31
Smoking	
Current	19
Former	37
None	56
Tumor Stage	
T1	17
T2	43
T3	27
T4	19
Ta	6
Classification	
Upper tract	31
Bladder	81
Sample Type	
Blood	34
Tumor	78

### Somatic alterations in Chinese UC patients

**Fig. 1:**
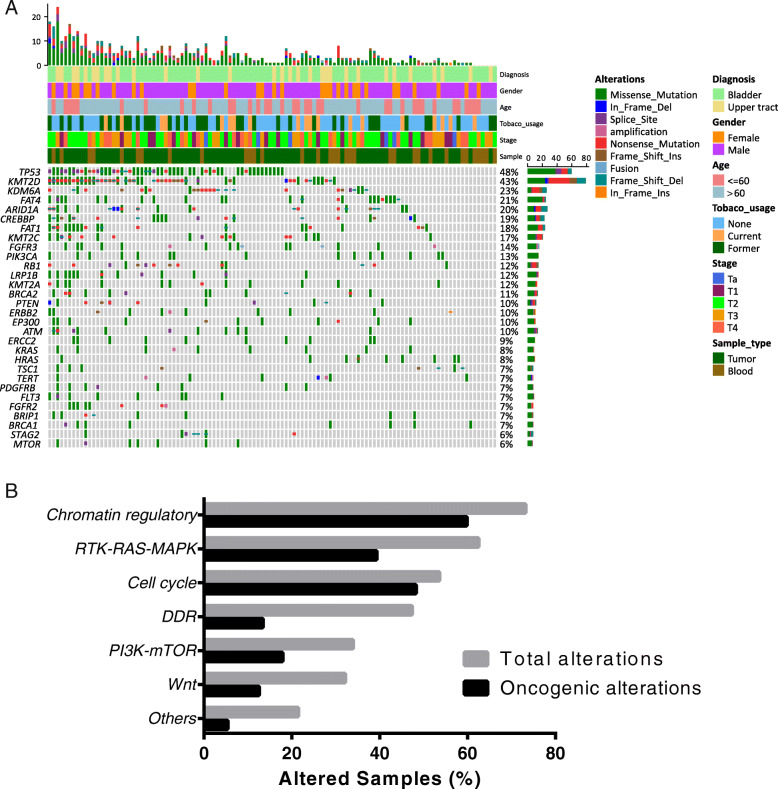
**a** Oncoprint representation of the 92 genes in 112 UC patients. Alterations were categorized as missense mutations (green), in frame deletion (dark blue) or insertion (orange), frameshift deletion (grey blue) or insertion (brown) and nonsense mutation (red). **b** The distribution of genomic alterations in the identified pathways

In total, 106 of 112 UC samples (94.64%) had valid somatic alterations. The mean and median counts of somatic alterations per sample were 6.05 and 4, respectively. Six samples did not contain any valid somatic mutations after excluding the germline alterations. The most frequently mutated genes in our cohort were *TP53* (48%), *KMT2D* (43%), *KDM6A* (23%), *FAT4* (21%), *CREBBP* (20%), *ARID1A* (19%) and *FAT1* (18%), respectively (Fig. 1a). Notably, 62.50% (70/112) and 86.61% (97/112) patients in our cohort were identified as having actionable and oncogenic alterations according to the OncoKB database, respectively [7]. No significant difference was found in the prevalence of genetic alterations between low- and high-grade urothelial carcinoma. Pathway analysis indicated that the most frequently enriched pathways were Chromatin regulatory (73.21%), RTK-RAS-MAPK (62.50%), cell cycle (53.57%), DNA damage repair (DDR, 47.32%), PI3K-mTOR (33.93%) and Wnt (32.14%), respectively (Fig. 1b).

### Germline alteration

After excluding the variants identified as benign or likely benign according to the American College of Medical Genetics and Genomics (ACMG) guideline, 244 germline variants were identified, and 75.89% (85/112) of patients in our cohort harbored at least one germline alteration (Supplemental Table 3). However, only two patients (1.85%) had variants that could be classified as pathogenic or likely pathogenic (*ERCC4* p.Lys481fs and *BRCA2* p.Thr3030fs). The patient harbored a deleterious *BRCA2* germline variant was also concurrent with prostate cancer. None of pathogenic or likely pathogenic germline variant associated with Lynch syndrome, including in *MLH1*, *MSH2*, *MSH6* and *PMS2* genes, was identified in any UTUC or UC patient in our cohort.

### Differences of somatic gene alterations in UC patients between our cohort and Western cohort

**Fig. 2:**
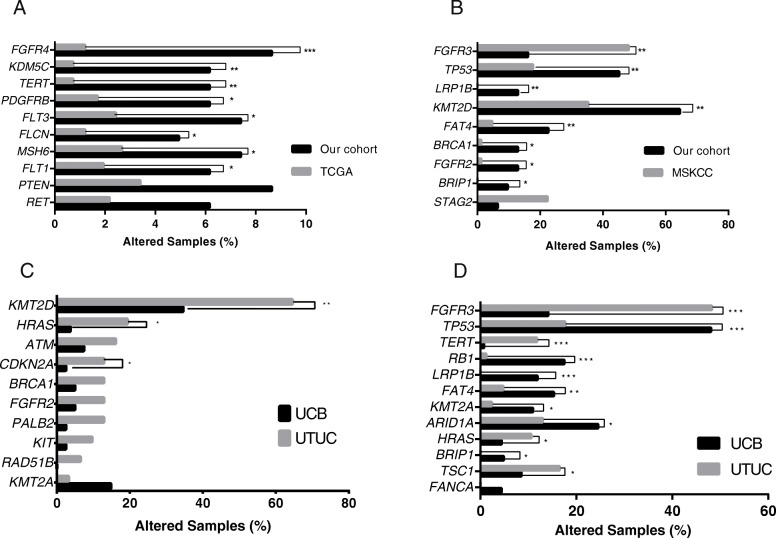
**a** Comparison of the prevalence of altered genes identified in UCB between our cohort (black bars) and TCGA database (grey bars). **b** Comparison of the prevalence of altered genes prevalence identified in the UTUC between our cohort (black bars) and MSKCC cohort (grey bars). Comparison of the prevalence of altered genes identified between the UCB and UTUC in our cohort (**c**) and in western cohort (**d**). Two-sided Fisher’s tests were conducted to compare the different frequency between two groups. UCB: urothelial carcinoma of bladder; UTUC: upper tract urothelial carcinoma. ***p≤ 0.001, **p≤ 0.01, *p≤ 0.05

To determine the potential difference of genomic features between Western and Chinese UC patients, we compared the alterations data of the selected 92 genes between our cohort and Western cohorts (UCB data published by TCGA and UTUC data published by MSKCC). The prevalence of alterations in *FGFR4*, *KDM5C*, *TERT*, *PDGFRB*, *FLT3*, *FLCN*, *MSH6*, *FLT1* were higher in the 81 UCB cases in our cohort, compared with the TCGA cohort (Fig. 2a). As for UTUC, a higher frequency of genetic alteration, including in *TP53*, *LRP1B*, *KMT2D*, *FAT4*, *BRCA1*, *FGFR2* and *BRIPI*, were found in our cohort of 31 cases compared with the MSKCC cohort (Fig. 2b). On the contrary, the prevalence of *FGFR3* alteration in the MSKCC cohort was three times higher than that in our cohort (48.24% versus 16.13%, *p* < 0.001).

### Differences of somatic gene alterations between UTUC and UCB

To elucidate the differences of somatic alterations between UTUC and UCB, we compared the alterations from 81 UCB samples and 31 UTUC samples in our cohort. The genomic features of UCB and UTUC were similar, while *KMT2D* (34.57% vs 64.52%, *p* < 0.01), *HRAS* (3.7% vs 19.35%, p < 0.01) and *CDKN2A* (2.47% vs 12.9%, *p* < 0.05) were significantly more mutated in UTUC than in UCB (Fig. 2c). A higher mutated frequency of *HRAS* was also presented in UTUC compared with UCB in Western cohorts, but with the additionally significant difference in the prevalence of *FGFR3*, *TP53*, *TERT*, *RB1*, *LRP1B*, *FAT4, KMT2A*, *ARID1A*, *BRIP1* and *TSC1* (Fig. 2d).

### Genomic alterations in *FGFR* genes

**Fig. 3:**
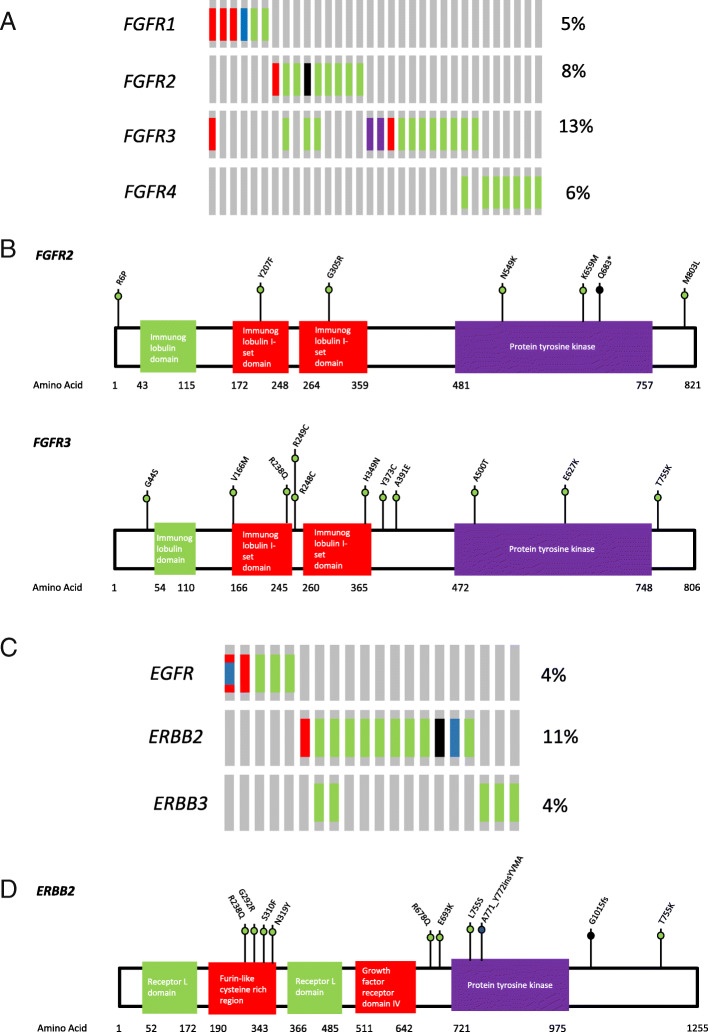
Genomic features of FGFR and ERBB alterations in UC patients. **a** Oncoprint illustration of somatic alterations in FGFR genes (*FGFR1, FGFG2, FGFR3* and *FGFR*4). **b** Alterations of *FGFR2* and *FGFR3* genes and amino acid changes identified in the study cohort. **c** Oncoprint illustration of somatic alterations in ERBB genes (*EGFR*, *ERBB2* and *ERBB3*). **d** Alterations of *FGFR2* and *FGFR3* genes and amino acid changes identified in the study cohort. Red: amplification; Blue: inframe insertion or deletion; Green: Missense; Black: Truncating

Although 28.57% (32/112) UC cases in our cohort had at least one somatic alteration in FGFR genes (including *FGFR1*, *FGFR2*, *FGFR3* and *FGFR4*), only 11 cases contained alternations that could be annotated as gain of function (Fig. 3a). Notably, six of them had multiple oncogenic alterations in FGFR genes. In line with previous studies, the most frequently altered gene was *FGFR3* (13.39%). Hotspot variants, including *FGFR3*-p.Ser249Cys, p.Arg248Cys and p.Tyr373Cys were present in six samples, while *FGFR3*-TACC3 fusion was only identified in two patients. Three novel variants (defined as unreported in literature or SNP databases and without ExAC frequency), including p.His349Asn, p.Val166Met and p.Thr755Lys of *FGFR3* were identified, though their functions were still unknown (Fig. 3b). Nine patients (8.04%) were identified with *FGFR2* alterations, of which only three alterations could be defined as oncogenic, including one p.Asn549Lys, one p.Lys659Met and one copy number gain (32.87 copies). No *FGFR2* fusion was identified. Four novel variants with unknown function, including p.Gly305Arg, p.Tyr207Phe, p.Met803Leu and p.Gln683Ter in *FGFR2* were identified in this study. Only six patients carried *FGFR1* gene alterations, and three of them were amplification. Eight patients (all were bladder cancer) carried nonsynonymous single nucleotide variants with unknown function in *FGFR4*.

### Genomic alterations in ERBB family

Twenty of our cases (17.86%) carried somatic alterations in the ERBB genes (*EGFR, ERBB2* and *ERBB3*), and three of them had dual alterations (Fig. 3c). Five patients (4.46%) carried *EGFR* gene alteration and two of them had EGFR copy number gain. Notably, one patient had an activated *EGFR* exon 20 insertion (*EGFR*-p.Val769_Asp770insAspAsnPro) and copy number gain. We observed *ERBB2* alterations in 12 patients (10.71%), and most of the oncogenic alterations were located in furin-like cysteine rich region (amino acid 190–343) and protein tyrosine kinase domain (amino acid 721–975) (Fig. 3d). Two of the five identified *ERBB3* alterations were in the furin-like cysteine rich region (amino acid 182–332) and oncogenic.

### Somatic alterations in DNA repair pathway

**Fig. 4:**
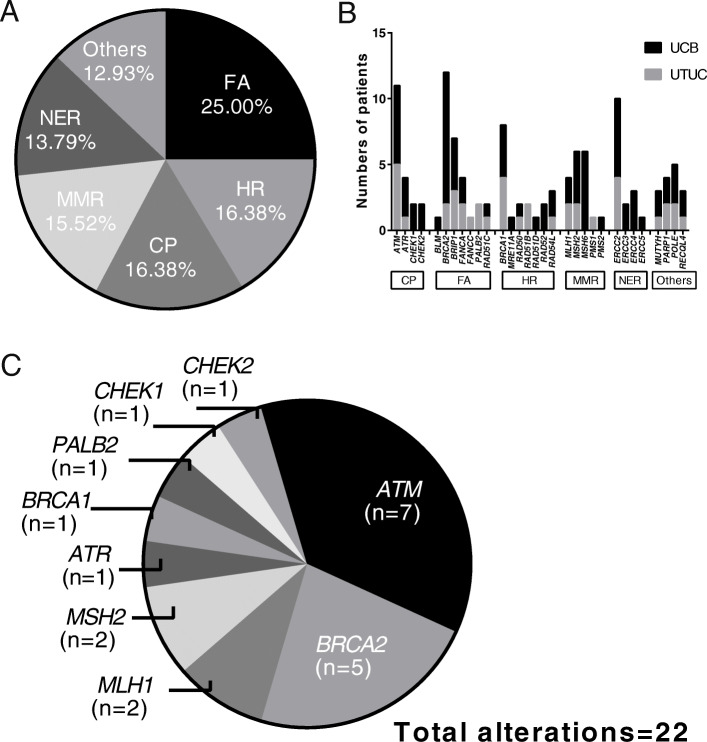
Somatic alterations in DDR pathway. **a** Frequency of altered pathway for DDR. **b** Distribution of somatic alterations in specific DDR gene of UTUC and UCB samples. **c** The distribution of known or likely deleterious somatic DDR gene mutations. DDR: DNA damage repair; UCB: bladder carcinoma; UTUC: upper tract urothelial carcinoma; FA: Fanconi anemia; HR: homologous recombination repair; CP: checkpoint; MMR: mismatch repair; NER: nucleotide excision repair

We observed 47.32% of cases in our cohort had alterations in the DDR pathway as defined by the MSKCC DDR gene panel [8], including 37 (45.68%, 37/81) UCB patients and 16 (51.61%,16/31) UTUC patients. The distribution of alterations among the six specific DDR pathways was relatively even, with a slight enrichment in the Fanconi anemia (FA) pathway (Fig. 4a). The most frequently mutated DDR genes were *BRCA2* (10.71%), *ATM* (9.82%), *ERCC2* (8.93%), *BRCA1* (7.14%) and *BRIP1* (6.25%), respectively (Fig. 4b). Among patients who carried DDR gene alterations, only 16 of them (14.29%) had at least one known or likely deleterious somatic DDR alterations (Fig. 4c), and the most frequently mutated DDR genes with known or likely deleterious variants were *ATM* (*n* = 7, 31.82%) and *BRCA2* (*n* = 5, 22.73%). Eighteen patients (16.07%) carried somatic alterations in DNA mismatch repair (MMR) genes, including in *MLH1*, *MSH2*, *MSH6* and *PMS2*. The majority of these MMR gene alterations were unknown of function, and only four alterations in *MSH2* and *MLH1* could be annotated as loss of function.

### Concordance of genetic alterations between ctDNA in plasma and matched tumor tissue

**Fig. 5:**
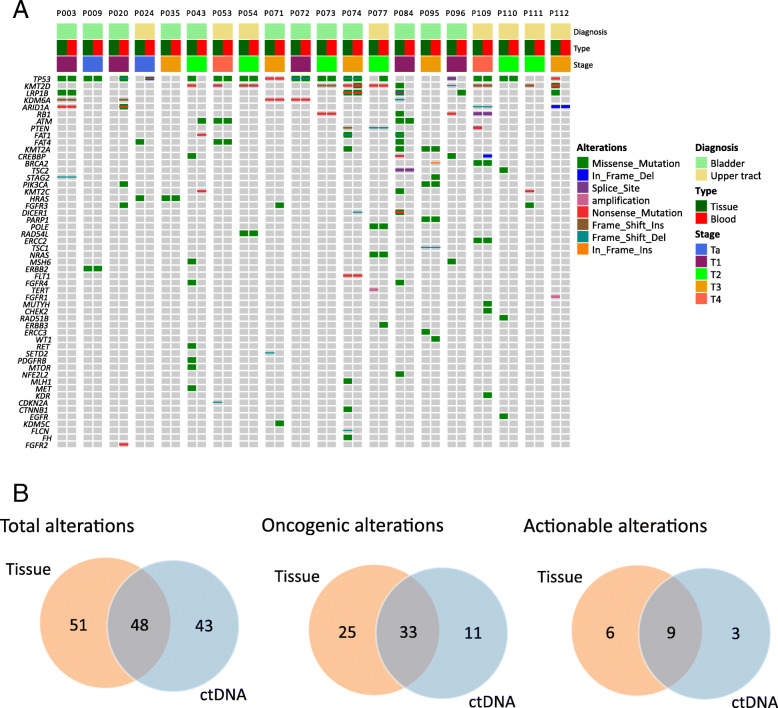
Concordance of genetic alterations between ctDNA in plasma and matched tumor tissue. **a** Oncoprint illustrating of the matched plasma and tumor samples. **b** Venn diagrams showed the number of total mutations, oncogenic mutations and actionable mutations in the matched plasma and tumor samples

Of the enrolled 112 patients, twenty of them provided matched tumor tissue and blood samples to comprehend the concordance of genomic alterations between different sample types in UC. The cfDNA abundance and ctDNA fraction was calculated according to the method described by Annala Matti, et al. [9]. Only three patients’ matched samples were collected with an interval time of more than 1 month (7 months for P074, 18 months for P084 and 34 months for P110, respectively). In total, 99 and 91 somatic alterations were identified in tumor and matched plasma ctDNA samples, respectively. Only one patient’s ctDNA was negative but the matched tumor sample were positive for alterations. Conversely, in Patient 20, ctDNA analysis revealed oncogenic mutations in *PIK3CA*, *TP53*, *ARID1A* and *KDM6A* with allele fractions beyond 3%, but no corresponding valid alteration was found in the matched tissue sample (Fig. 5a). There was no significant difference between tissue and blood in the median number of genomic alterations (4 versus 4, *p* = 0.7436). By comparison of blood and matched tumor tissue, the overall concordances for genomic alterations and altered genes identified in matched samples were 42.97% (0–100%) and 46.83% (0–100%), respectively (Table 2). Among the genomic alterations, 48 of them were in concordance between ctDNA and tumor tissue (Fig. 5b, left). Fifteen actionable alterations were identified in tissue samples as defined by the OncoKB database, and 60% of them were shared by the matched ctDNA (Fig. 5b, middle). Notably, 43 of 91 (47.25%) alterations detected in ctDNA were not detected in the corresponding tissue sample. Specifically, 11 of them (25.58%) were oncogenic mutations and 3 (6.98%) could be identified as actionable, including a *PIK3CA* activated hot-spot, a *PTEN* frameshift and a *KDM6A* frameshift mutation.

**Table 2:** Genomic alterations identified in matched tissue and blood

Patient number	Genomic alterations in tissue, number	Genomic alterations in blood, number	Genes altered in both samples, number (%)	Genomic alterations in both samples, number (%)
P003	6	5	5 (83.3%)	5 (83.3%)
P009	2	7	2 (28.57%)	2 (28.57%)
P020	0	10	0	0
P024	2	2	0	0
P035	1	1	1 (100%)	1 (100%)
P043	4	3	0	0
P053	5	9	5 (62.5%)	3 (27.27%)
P054	4	4	4 (100%)	4 (100%)
P071	3	4	2 (50%)	2 (40%)
P072	3	3	3 (100%)	3 (100%)
P073	3	3	3 (100%)	3 (100%)
P074	15	10	4 (36.36%)	6 (30%)
P077	5	5	4 (66.67%)	4 (66.67%)
P084	17	4	2 (13.33%)	2 (9.52%)
P095	4	7	4 (57.14%)	4 (57.14%)
P096	4	1	0	0
P109	9	11	7 (63.64%)	7 (63.64%)
P110	3	1	1 (50%)	1 (33.33%)
P111	4	0	0	0
P112	5	1	1 (25%)	1 (20%)

## Discussion

Compared with UC patients in Western countries, more Chinese UC patients were diagnosed in the advanced stage for multiple reasons [10]. Meanwhile, fewer drugs were developed and approved in China for advanced UC, which left no choice but only chemotherapy for most Chinese patients with advanced UC had no other treatment choice but only chemotherapy. However, because of more possibility of aristolochic acid exposure, many Chinese UC patients, especially UTUC, tend to have chronic kidney disease as well, which made them unfit for platinum treatment [5]. Therefore, it’s important to understand the genomic features of Chinese UC patients for therapy development. Remarkably, over 60% of UC patients in this study were identified to have at least one potentially actionable somatic alteration, which may offer more therapeutic intervention chances.

Currently, activating mutations in *FGFR2* and *FGFR3* genes were actionable with highest level of evidence in UC, especially for UTUC, as erdafitinib (a *FGFR* inhibitor) was proved to have a 40% objective response rate in previously treated locally advanced and unresectable or metastatic UC with *FGFR*2/3 alterations [11]. We identified *FGFRs* alterations in 26.85% of UC patients, which was close to the corresponding ratio reported in the TCGA database [4]. Although they may be associated with increased sensitivity to FGFR inhibitors, UC patients with *FGFR* alterations were reported to have a lower response rate to the ICIs therapy because of the suppressed infiltration of immune cells in the tumor microenvironment [12, 13]. Alterations in ERBB pathway, especially *ERBB2/HER2* in UC, raised increasing research interest recently, as 12.4 and 11% UCB patients were reported to have ERBB2 overexpressing (as shown by positive HER2 immunohistochemistry staining) or activating mutations, respectively [14]. Similarly, ERBB2 alteration was observed in 10.71% of cases in our cohort, which was similar to the data in Western UC patients [4]. An interesting correlation between *ERBB2* gene mutations and a higher probability in response to platinum-based neoadjuvant chemotherapy was found in 61 muscle-invasive UCB [15]. Meanwhile, Trastuzumab combined with chemotherapy was proved to have an outstanding response rate of 70% and a median overall survival of 14.1 months in 44 advanced UC patients with HER2/ERBB2 overexpression in a single-arm phase II trial [16]. Other anti-HER2 therapies, such as tyrosine kinase inhibitors, antibodies and antibody-drug conjugate, had been under development for UC patients with abnormal *ERBB2* [17].

DDR alterations had been reported to be associated with a higher response rate and increased clinical benefit for immune checkpoint inhibitors (ICIs), platinum-based neoadjuvant therapy and first-line chemotherapy in UC patients [18]. A previous study found 23.9% UCB patients had alterations in homologous recombination repair gene pathway [19]. In our study, we found 47.32 and 14.29% UC patients had alterations and deleterious alterations in DDR pathway, respectively. The ratios were consistent with the previous study analyzing the same panel of the genes [8]. In addition to ICIs, tumors with deleterious DDR gene mutations, especially BRCA1/2 genes, were also associated with higher sensitivities to poly (ADP ribose) polymerase inhibitors (PARPi) in pan-cancers [20]. Though the proved efficacy of PARPi in UC patients is still limited, PARPi combined with ICIs has been demonstrated to have cooperative effects in treating UC patients with HR mutations [21].

The prevalence of germline mutations of cancer susceptibility genes in patients with sporadic UC was conflicting. Although previous studies established that UC was rarely associated with cancer susceptibility genes alterations, the latest research found that 14% of 586 unselected UC patients carried pathogenic or likely pathogenic germline variants, and 11.26% (66/586) had deleterious variants in the DNA repair pathway [22]. Until now, the prevalence of germline mutations of cancer susceptibility genes in Chinese UC patients remains unknown. In our cohort, only 1.79% of patients had pathogenic or likely pathogenic germline variants, which may represent a low prevalence of cancer susceptibility genes in Chinese UC patients compared to the corresponding Caucasian patients. In this study, we found a novel likely-pathogenic *ERCC4* gene germline variant in a UTUC patient. Previously, *ERCC4* gene mutations were identified in Fanconi anemia, skin-photosensitive nucleotide excision repair (NER)-deficient disorder xeroderma pigmentosum, and XFE progeroid syndrome [23]. Somatic alterations in *ERCC4* have been detected in 2.03% of UC patients, yet no carrier for deleterious germline ERCC4 mutations have been reported in UC [24]. Our study is the first to report germline mutations in *ERCC4.* Moreover, previous research found that 7–8.3% unselected UTUC patients had Lynch syndrome-related features, such as deficient mismatch repair (dMMR), microsatellite instability high (MSI-H) or deleterious alterations of MMR genes, while only 2.1% occurred in UC patients [22, 25]. In the present research, no germline variant in mismatch repair pathway was found, potentially due to the different genetic backgrounds between Chinese and Western UC patients, limited UTUC patients enrolled and technology limitations (such as lack of detecion in MLH1 promoter hypermethylation and large rearrangements) [26].

ctDNA has been reported as a biomarker in cancer diagnosis, risk stratification, treatment evaluation and relapse monitoring in UC [27]. However, several studies published controversial results about the concordance between tissue and blood in other genitourinary carcinomas [28]. We found a concordance of 42.97% (0–100%) of the genetic alterations between 20 ctDNA and matched tissue samples. To date, there is no published study reported the concordance of genomic alterations between matched ctDNA and tumor tissue based on the same testing panel. Comparison of ctDNA and tumor tissues in 22 metastatic UC patients in different testing panel (FoundationOne for tissue and Guardant360 for ctDNA, respectively), the overall concordance was only 16.4–17.1% [29]. Agarwal et al. reported ctDNA could mimic the feature of some biomarkers in tumor tissue, however, this conclusion was achieved by the comparison of the unmatched results between samples in their cohort and TCGA database, without overall concordance analysis [30]. Though multiple factors, including tumor stage, metastasis status, treatments, the interval time between sample collection and testing panel would affect the concordance of genomic alterations between ctDNA and tissue, our results demonstrated that liquid biopsy was still an expanding way to identify gemomic alterations in UC patients. Further studies with larger sample size may be need to investigate the application of ctDNA in UC patients.

## Conclusion

In this study, we identified a unique genomic feature of germline and somatic alterations in Chinese UC patients by next-generation sequencing. In total, 62.50% of 112 Chinese UC patients had at least one actionable genomic alteration, which would benefit from matched or related target therapies. Furthermore, our study found a reasonably good concordance between ctDNA and matched tumor, suggesting the potential application of liquid biopsy in UC.

## Supplementary Information

**Additional file 1.**

**Additional file 2.**

**Additional file 3.**

## Data Availability

The raw sequencing data files were deposited in the Chinese National Genomics Data Center (https://bigd.big.ac.cn/gsa-human/browse/HRA000491). Left biospecimens may be shared for academic research under a prior approval from the government of China.

## Abbreviations

UC: Urothelial Carcinoma
ctDNA: Circulating Tumor DNA
cfDNA: Cell-free DNA
gDNA: Germline DNA
UTUC: Upper Tract Urothelial Carcinoma
UCB: Urothelial Carcinoma of Bladder
ICI: Immune Checkpoint Inhibitor
TCGA: The Cancer Genome Atlas
MSKCC: Memorial-Sloan Kettering Cancer Center
FGFR: Fibroblast Growth Factor Receptor
ERBB: Erythroblastic Leukemia Viral Oncogene Homolog
DDR: DNA damage repair
